# PELP1 Suppression Inhibits Gastric Cancer Through Downregulation of c-Src-PI3K-ERK Pathway

**DOI:** 10.3389/fonc.2019.01423

**Published:** 2020-02-13

**Authors:** Hongzhu Yan, Yanling Sun, Qian Wu, Zhe Wu, Meichun Hu, Yuanpeng Sun, Yusi Liu, Zi Ma, Shangqin Liu, Wuhan Xiao, Fuxing Liu, Zhifeng Ning

**Affiliations:** ^1^Basic Medical School, Hubei University of Science and Technology, Xianning, China; ^2^Institute of Basic Medical Sciences, Hubei University of Medicine, Shiyan, China; ^3^Wuhan University Zhongnan Hospital, Wuhan, China; ^4^The Key Laboratory of Aquatic Biodiversity and Conservation, Institute of Hydrobiology, Chinese Academy of Sciences, Wuhan, China

**Keywords:** PELP1, oncogene, master gene, gastric cancer, c-Src-PI3K-ERK pathway, chlorpromazine, therapeutic target

## Abstract

**Background:** Proline-, glutamic acid-, and leucine-rich protein 1 (PELP1), a co-activator of estrogen receptors alpha, was confirmed to be directly associated with the oncogenic process of multiple cancers, especially hormone-dependent cancers. The purpose of our research was to explore the biological function, clinical significance, and therapeutic targeted value of PELP1 in gastric cancer (GC).

**Methods:** The expression status of PELP1 in GC cell lines or tissues was analyzed through bioinformatics data mining. Thirty-six GC tissue chip was applied to demonstrate the results of bioinformatics data mining assayed by immunohistochemical method. The expression status of PELP1 in GC cell lines was also analyzed using western blot. Correlation analysis between PELP1 expression and clinicopathological parameter was performed. Kaplan-Meier survival analysis was applied to analyze the relationship between PELP1 expression and total survival time. Three pairs of siRNA were designed to silence the expression of PELP1 in GC. After PELP1 was silenced by siRNA or activated by saRNA, the growth, plate colony formation, migration and invasion ability of the GC cell or normal gastric epithelium cell line was tested *in vitro*. Cell cycle was tested by flow cytometry. Nude mice xenograft experiment was performed after PELP1 was silenced. The downstream molecular pathway regulated by PELP1 was explored. Molecular docking tool was applied to combine chlorpromazine with PELP1. The inhibitory effect of chlorpromazine in GC was assayed, then it was tested whether PELP1 was a therapeutic target of chlorpromazine in GC.

**Results:** PELP1 expression was elevated in GC cell lines and clinical GC tissue samples. PELP1 silence by siRNA compromised the malignant traits of GC. PELP1 expression positively correlated with tumor invasion depth, lymph node metastasis, tissue grade, TNM stage, but had no correlation with patient age, sex, tumor size, and tumor numbers. Kaplan-Meier survival analysis revealed high PELP1 expression had a shorter survival period in GC patients after follow-up. Q-PCR and western blot revealed PELP1 suppression in GC decreased expression of the c-Src-PI3K-ERK pathway. It was also implied that chlorpromazine (CPZ) can inhibit the malignant traits of GC and downregulate the expression of PELP1.

**Conclusions:** In a word, PELP1 is an oncogene in gastric cancer and c-Src-PI3K-ERK pathway activation may be responsible for its tumorigenesis, PELP1 may be a potential therapeutic target of chlorpromazine in GC.

## Introduction

Gastric cancer (GC), the fifth most common form of cancer worldwide, is a leading cause of cancer-related death (1). In China, based on the aging, unhealthy lifestyle, such as ingesting too much-salted product, smoking, and heavy drinking, the high infection rate of Helicobacter pylori, the incidence of GC ranks the second (2), with a plenty advanced GC duing to insidious and unspecific symptom. Progressive clarification of pathogenesis and improvement of therapeutics seems not to improve the prognosis of GC patients, 5-year survival rate and total survival time hover at a low level. Recurrence and metastasis still are responsible for the death of GC patients. At present, there is no effective comprehensive treatment to cure advanced GC. Therefore, it is important to determine the molecular mechanisms that lead to GC invasion and metastasis so as to exploit new therapeutic target and drugs.

Proline-, glutamic acid-, and leucine-rich protein 1 (PELP1) is a modulator of non-genomic actions of estrogen receptors (NMAR) and a coregulator of estrogen receptor; PELP1 has been implicated in many physiological (3–5) and pathological processes (6). Research has demonstrated that PELP1 is a proto-oncogene in all hormone-responsive cancers, such as breast (7, 8), ovarian (9), endometrial (10), and prostate cancers (11, 12). Moreover, some researchers determined the oncogenic functions of PELP1 in hormone-non-responsive cancers, such as brain tumor (13), lung cancer (14), and colorectal cancer (15).

In the present study, we explored the functions of PELP1 in the malignant biologic traits of GC. Bioinformatics and western blot showed that PELP1 expression was higher in GC cell lines than in normal gastric epithelium. PELP1 silence by small interfering RNA (siRNA) inhibited proliferation, colony formation, migration, invasion, and promoted the apoptosis of the GC cell line AGS. On the contrary, PELP1 activation by small activating RNA incurred proliferation, colony formation, migration, invasion of immortal gastric epithelium cell line GES-1. Meanwhile, PELP1 silencing was concomitated by the c-Src-PI3K-ERK pathway downregulation. Importantly, antipsychotic drug chlorpromazine can suppress the malignant traits of GC through the downregulation of PELP1. Our study of the PELP1 signaling pathway may contribute to a better understanding of GC invasion and metastasis, providing independent prognostic indicators and therapeutic targets for GC.

## Materials and Methods

### Cell Lines and Cultures

Human gastric adenocarcinoma cells (AGS, SNU-520 and SNU-1) were purchased from the Type Culture Collection of the Chinese Academy of Science (Shanghai, China), human gastric epithelial cells (GES-1 cell) was purchased from American Type Culture Collection (Manassas, VA, USA). All cells were cultured in RPMI1640 medium mingled with 10% fetal bovine serum (Gibco), 100 Unit/ml penicillin and 100 μg/ml streptomycin. All the cells were cultured at 37°C and saturated humidity cell incubator filled with 5% CO_2_.

### Antibodies and Reagents

The antibodies used were explained in the western blot experiment. Transfect reagent Lipofectamine 2000^TM^ was purchased from Thermo Scientific company. Three pairs of siRNA were designed using the free online tool in the website of Ambion and synthesized by Genephama company in China. The siRNA sequence was listed in Table 1. Other reagents were purchased from Gibco or Sigma Aldrich company except for specific instructions.

**Table 1:** siRNA sequence.

**Name**	**Sense (5**′**-3**′**)**	**Antisense (5**′**-3**′**)**
Negative control	UUCUCCGAACGUGUCACGUTT	ACUUGACACGUUCGAAGAATT
siRNA1	GUCAGUAGCAAGAAUAUUATT	UAAUAUUCUUGCUACUGACTT
siRNA2	GGCUUGUGGUUCUCUCAAATT	UUUGAGAGAACCACAAGCCTT
siRNA3	CAAGGUGUAUGCGAUAUUATT	UAAUAUCGCAUACACCUUGTT

### Molecular Docking Modeling Assay

The structures of the ligands were built and energy optimized using the Modeller9.10 software. We used free AutoDock Toolkit developed by the Scripps Research Institute and Olson lab for docking experiments. All of the water molecules were removed before the experiments so that our experiments were performed under non-aqueous conditions. The primary ligand bound to the binding pocket was the chosen conformation for the active site. The picture was prepared using Pymol 1.2R2 version.

### Bioinformatics

The mRNA expression of PELP1 in GC tissues or cell lines and paired adjacent gastric mucosa tissues or epithelium was acquired from Oncomine (http://www.oncomine.org) and CCLE(Cancer Cell line Encyclopedia). The pathway information regulated by PELP1 was obtained from BioCarta (https://cgap.nci.nih.gov/Pathways/BioCarta_Pathways).

### saRNA Activation of PELP1

saRNA was designed according to previous report performed by Li et al. In brief, the promoter sequence of human PELP1 gene was extracted from NCBI website. Three pairs of double strand RNA, 21 nucleotide in length, were designed to match the promoter sequence of PELP1. saRNA sequence was listed in Table 2. saRNA was synthesized in GenePharma company in China, then transfected into gastric epithelium cell line GES-1 through transfectant reagent Lipofectamine 2000^TM^. Activation effect was assessed by western blot.

**Table 2:** saRNA sequence.

**Name**	**Sense (5**′**-3**′**)**	**Antisense (5**′**-3**′**)**
Negative control	ACUUACGAGUGACAGUAGA[dT][dT]	UCUACUGUCACUCGUAAGU[dT][dT]
saPELP1-142	AGAGCGAGACUCCGUCUCA[dT][dT]	CCUCGCUCUGAGGCAGAGU[dT][dT]
saPELP1-191	AGGUUGCGGUGAGCCGAGA[dT][dT]	UCCAACGCCACUCGGCUCU[dT][dT]
saPELP1-247	UAAUCCCAUCUACUCGGGA[dT][dT]	AUUAGGGUAGAUGAGCCCU[dT][dT]

### Immunohistochemical Analysis

Gastric cancer tissue microarray (TMA) were obtained from GugeBio in China. This array contained 72 tissues isolated from gastric cancer patients, including thirty-six gastric cancer specimens and corresponding adjacent normal gastric mucosa tissues. Immunohistochemical staining using a polyclonal antibody against PELP1 (A13414, 1:100 for IHC analysis, ABclonal) was performed. After staining, the tissues were used for further data analysis. PELP1 expression in TMA of cancer and adjacent tissues was performed by microscopic analysis. Briefly, after IHC staining, if cells or tissues are stained browns from light yellow, they are recorded as positive immunostaining. Concretely, for tumor tissue, cancer analysis software (Media Cybernetics, Inc. Silver Spring, MD USA). The average densitometry of the digital image (50 μm) was designated as the representative PELP1 staining intensity (representing relative PELP1 expression level). The signal densities from the tissue regions of the five randomly selected visions were blindly counted and statistically analyzed. Correlation of clinicopathological data of above 36 gastric cancer patients and positive PELP1 expression was analyzed. Also, the relationship between PELP1 expression and survival period was explored.

### Western Blot

The extraction of total protein was using the RIPA buffer (1% Triton X-100, 50 mM Tris-HCl pH 7.2, 0.1% SDS and 1 mM EDTA, 150 mM NaCl, 1% sodium deoxycholate). Using the bicinchoninic acid method to quantify, 20–25 μg protein was separated by sodium dodecyl sulfate-polyacrylamide gel electrophoresis (SDS-PAGE). Moreover, the protein was transferred to 0.45 μM nitrocellulose membranes. Membranes were soaked in TBS-T buffer containing 5% bovine serum albumin for about 1 h and incubated with the following primary antibodies (rabbit anti-PELP1 antibody, A3189, 1:1,000, ABclonal) overnight at 4°C. anti-c-Src antibody (A0324, 1:1,000, ABclonal), anti-Phospho-Src-Y529-antibody (AP0185, 1:500, ABclonal) and mouse polyclonal GAPDH antibody (SC-47724, 1:1,000, Santa Cruz). After washing with TBS-T, the membrane was then conjugated with horseradish peroxidase (HRP) goat anti-rabbit IgG antibody (1:5,000, Proteintech) or goat anti-mouse IgG antibody (1:20,000; Proteintech) at room temperature Hybridization for 1 h. The blots were photographed by the Fujifilm LAS4000 mini luminescent image analyzer, and the density of the protein bands was quantified by Multi Gauge V3.0 analyzer software (FujiFilm Corp., Tokyo, Japan).

### Quantitative Real-Time PCR (qRT-PCR)

The method of RNA extraction and quantitative real-time PCR (qRT-PCR) according to the protocol previously described (16). Total RNA was extracted from the cells by Trizol reagent, and then cDNA was synthesized. Subsequently, using the QuantiTectSYBR® GreenRT-PCR kit to perform qRT-PCR. Relative expression values were calculated using the 2-ΔΔCt method. GAPDH was used as interns control. The sequence of specific primers is listed in Table 3.

**Table 3:** Primers for Q-PCR.

**Gene**	**Forward primers**	**Reverse primers**
GAPDH	5**′**-AGCCACATCGCTCAGACAC-3**′**	5**′**-GCCCAATACGACCAAATCC-3**′**
PELP1	5**′**-GGAAGATGGCGGCAGCCGTT-3**′**	5**′**-TCACTGCCGAGAGACCCCCG-3**′**
c-Src	5**′**-CTCTTCAGAGCCCTTGCTCA-3**′**	5**′**-ATTCACCCTCCCCCAAGGAA-3**′**
PI3K	5**′**-AACGAGAACGTGTGCCATTTG-3**′**	5**′**-AGAGATTGGCATGCTGTCGAA-3**′**
Erk	5**′**-CCAGACCATGATCACACAGG-3**′**	5**′**-CTCGTCACTCGGGTCGTAAT-3**′**

### Cell Transfection

PELP1-siRNA1, PELP1-siRNA2, PELP1-siRNA3, saPELP1-142, saPELP1-191, saPELP1-247 negative control were designed and purchased from GenePharma (Shanghai, China). The AGS or GES-1 cells at a density of 1 × 10^6^ per well were seeded in the 6-well plate and cultured in an incubator for 24 h in 5% CO_2_ at 37°C until the cell confluence arrived at 70–90%. Transfections were processed by Lipofectamine 2000 (Invitrogen, Carlsbad, CA) according to the specifications of the manufacturer. After 6-h transfection, the culture medium was replaced with complete medium. The PELP1-siRNA used are listed in Table 2.

### Cell Counting Kit-8

The expression of PELP1-siRNA or PELP1-saRNA on AGS cell or GES-1 proliferation can be reflected by Cell Counting Kit-8 kit (CCK-8) (Dojindo, Kumamoto, Japan). Briefly, cells were plated in 96-well plates at a concentration of 1 × 10^3^ per well and incubated for 24 h. Then transfecting for 48 h by PELP1-siRNA, add 10 μL CCK-8, and then incubated for 4 h more. The absorbance of 450 nm was measured by Molecular Devices i3x+MinMax (Molecular Devices, Austria).

### MTT

Cell viability and proliferation activity were determined with the MTT assay. CPZ medication groups or control groups were seeded in 96-well plates (Costar) at a density of 5,000 cells per well in complete medium (RPMI1640 supplemented with 10% FBS, 1% p/s) and then incubated for 12 h under standard conditions (37°C and 5% CO_2_). The total volume in each well was 200μL. From the next day to the seventh day, 20 μL of MTT (5 mg/mL) was added into each well. After an additional incubation for 4 h, the solution in each well was replaced with dimethyl sulfoxide (Sigma, USA) to solubilize formazan, the metabolic product of MTT. The plates were kept on a shaking mixer for 10 min to guarantee complete solubilization of formazan, and the optical density was recorded at 490 nm using a microplate reader. Results were expressed as means ± SD, and a growth curve was constructed. Data were analyzed by one way ANOVA with the *post-hoc* Tukey test applied for paired comparisons.

### Plate Colony Forming Assay

Colony forming ability was examined by the plate colony formation assay. Experimental groups or controls were seeded into six-centimeter plates (Costar) at a density of 50, 100, 200 cells per plate in complete medium and then incubated for ~7 days under standard conditions (37°C and 5% CO2). The cells were washed twice with phosphate-buffered saline (PBS) and then fixed with methanol for 15 min. After staining with 0.1% crystal violet for 20 min, the number of positive colonies with diameters exceeding 50 μm was counted under a light microscope with 100 × magnification. The colony forming rate was calculated by dividing the number of positive colonies by the total number of cells seeded.

### EdU Labeling Assay

AGS cells in the exponential growth phase were seeded onto coverslips in 6-well plates at a density of 1 × 10^6^ cells/well and grown overnight prior to transfection with PELP1-siRNA for 48 h. The cells were incubated with complete medium and 50 μM EdU for 2 h at 37°C. At the end of the treatment period, the coverslips were washed with ice-cold PBS and fixed in 4% formaldehyde polymerization for 30 min at room temperature. Moreover, the cells were treated with 100 μl Apollo staining solution for 30 min and subsequently washed in 0.4% Triton 100 thrice. After incubating with 100 μl Hoechst 33342 for 30 min, cells were washed with PBS three times. Fluorescent images were acquired using a fluorescence microscope at magnification ×200 and ×400. Labeled cells were counted in at least five fields of each coverslip.

### Flow Cytometry

To confirm cell cycle status, the content of nuclear DNA was detected by flow cytometry using propidium iodide staining. Cells were fixed in 70% ethanol at 4°C for 30 min. After dealing with phosphate buffered saline (PBS), the cells were stained with 0.1% Triton 100 (Sigma), 20 μg/ml RNase A (Sigma) and 10 μg/ml propidium iodide (Sigma) for 30 min and analyzed by FACScan flow cytometry.

### Wound Healing Experiment

To assay the migrative ability, 5 × 10^5^ cells were inoculated into six-well plate and cultured in incubator. Fourty-eight hours later, a wound was made by scratching the cells in the wells of six-well plate. After scratching, wounding healing status was recorded every 12 h.

### Transwell Invasion and Migration Assay

To observe transwell chamber migration assay, PELP1-siRNA transfected cells (1 × 10^6^) were plated in the upper chamber (24-well insert; pore size: 8 μm; BD Biosciences). And for the invasion assay, the bottom of the upper chamber was filled with Matrigel (BD Biosciences) for 3 h at 37°C. In both methods, cells were inoculated in the upper chamber in a serum-free medium filled with 500 μL RPMI 1640 medium supplemented with 10% FBS (GIBCO BRL, Grand Island, NY) as a chemoattractant. The cells were cultured for 16–24 h and the cells that did not migrate or invade the wells were removed by a cotton swab. Cells that migrated on the lower surface of the membrane were fixed and stained with a 0.1% crystal violet staining solution. Count the cells at the bottom of the membrane from five different microscope fields and calculate the mean.

### Nude Mice Xenograft Experiment

Male BALB/c (nu/nu) mice aged 6–8 weeks were obtained from the Experimental Animal Center of Hubei Province. The mice were housed and maintained in laminar flow cabinets under specific pathogen-free conditions according to the regulations and standards approved by the Animal Care and Ethics Committee of Hubei University of Science and Technology. To establish s.c. tumors, 5 × 10^6^ AGS-siRNA or AGS-control cells were resuspended in 200 μL of RPMI1640 serum-free medium and injected via an 18-gauge needle into the s.c. space of both flanks of the mice. Tumor progression was documented once weekly by measurements using calipers, and tumor volumes were calculated by the following formula: length × width × height × 0.52 (in mm). The mice were given ethane anesthesia and then euthanized by cervical dislocation.

### Statistical Analysis

Values (score or stained signal intensities) are expressed as means ± SEM (standard error of the mean). The average density of cancer and adjacent tissues was compared by Students' t-test. Fisher exact test was used to assess the significance of PELP1 positive expression in thirty-six clinical samples related to clinical data. T-test or Mann–Whitney U test was used to calculate the significance of paired and unpaired continuous variables. Kaplan-Meier survival analysis was performed and the difference was analyzed using the log-rank test. *P* < 0.05 was considered statistically significant. All data analysis and plotting were accomplished in SPSS 17.0 software.

## Results

### PELP1 Expression Was Upregulated Significantly in Human GC

**Figure 1:**
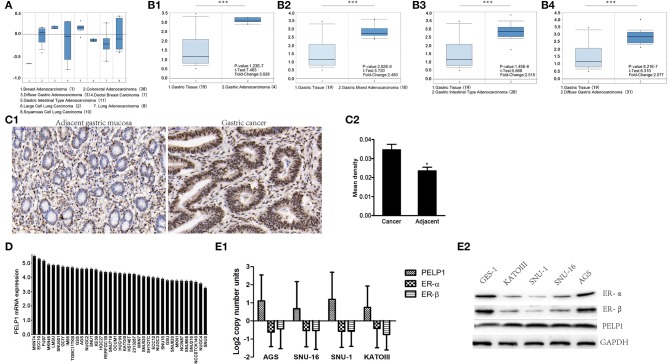
PELP1 expression was elevated in gastric cancer tissues and cells comparing with corresponding counterparts. **(A)** Box plots derived from gene expression data in ONCOMINE comparing expression of a specific PELP1 in different cancer tissues. **(B1–B4)** Oncomine data showed that PELP1 expression was elevated in GC tissues as compared with gastric normal tissues. **(C1)** Representative images of PELP1 level in normal gastric mucosa and GC specimens by immunohistochemistry. The positive rate of PELP1 in gastric cancer specimens was higher than in normal gastric mucosa. **(C2)**, PELP1 expression in normal gastric and GC tissues. The mean density of cancerous and adjacent gastric tissues from all 36 cases is illustrated at top graph. Difference was detected between the mean density of cancerous tissues (0.0346 ± 0.0172) and that of adjacent tissue (0.0235 ± 0.0115) using paired students' test, *p* < 0.05. **(D)** PELP1 was expressed in different stomach cell lines from CCLE. **(E1,E2)** The level of PELP1, ERα and ERβ was induced from Oncomine data base and validated by western bot in four GC cell lines and nomal gastric epithelium cell line GES-1. Mean ± SEM was applied for analysis (*p < 0.05, ***p < 0.001).

Public data (Oncomine) was utilized to assess the level of PELP1 in GC and adjacent normal tissues. PELP1 was significantly overexpressed in GC tissues relative to normal gastric mucosa (Figures 1A,B). Thereafter, we sought to verify the expression of PELP1 in 36 paired GC and surrounding normal tissues. Immunohistochemistry also found PELP1 was significantly higher in GC tissues (Figure 1C1). PELP1 immunohistochemistry results of expression in GC and its adjacent tissues were analyzed using Image-pro plus 6.0 software. The mean density of cancerous tissues from all 36 cases was 0.0346 ± 0.0172, while that of adjacent tissue was 0.0235 ± 0.0115 (data not shown). These results suggest a significantly higher level of PELP1 expression in cancerous tissues compared to their adjacent normal tissues (*p* < 0.05, Figure 1C2). Further analysis confirmed that PELP1 expression was positively correlated with tumor stage and grade; PELP1 expression was closely related to tumor differentiation (Table 4). Given other clinical parameters, our study showed that up-regulated PELP1 expression was closely associated with T and N staging, whereas no correlation was observed between PELP1 levels and gender or tumor size (Table 3). To further validate PELP1 expression in GC cell lines from the CCLE and Oncomine databases (Figures 1D,E1), expression of PELP1 was assayed in the AGS cell line and other GC cell lines such as SNU-1, SNU-16 and KATOIII compared to normal gastric epithelium cell line GES-1 using Western blot (Figure 1E2). We also tested the expression status of estrogen receptor α and β (ERα and ERβ). Astonished, both ERα and ERβ expression in GC was decreased compared with normal gastric epithelium GES-1 (Figure 1E2). The expression of PELP1 in AGS and SNU-1 cells were silenced to further investigate the effects of PELP1.

**Table 4:** Relationship between clinicopathological characteristic and PELP1 expression in gastric cancer.

**Clinicopathological characteristic**		**PELP1**		***P* value**
		**Negative**	**Positive**	
Gender	Male	8	12	0.503
	female	7	9	
T stage	T1–T2	7	1	0.046^*^
	T3–T4	8	20	
N stage	N0	8	6	0.01^**^
	N1–N2	7	15	
Grade	G1 well	5	5	0.017^*^
	G2 moderate	6	6	
	G3 poor	4	10	
Size	<5 cm	10	8	0.157
	≥5 cm	6	12	
pStage	I–II	8	7	0.014^*^
	III–IV	5	16	
Age	≤60	10	7	0.505
	>60	8	11	

* *p < 0.05*,

** *p < 0.01*.

### PELP1 Silencing Inhibited Growth, Migration, Invasion, and Xenograft of GC

**Figure 2:**
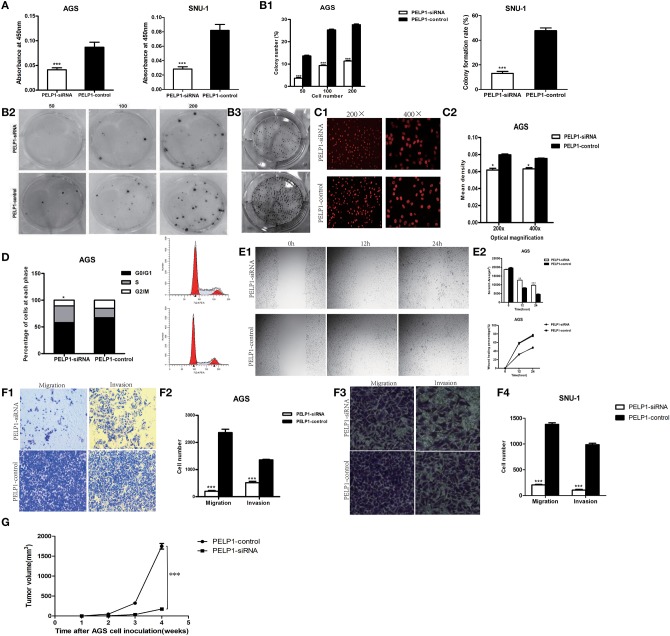
PELP1 knowdown inhibited proliferation, migration and invasion, xenograft of gastric cancer cell. **(A)** The absorbance (OD) at 450 nm wave-length was reduced in PELP1 knockdown group in CCK-8 assays. PELP1 silencing inhibited the colony formation ability of AGS **(B1)** and SNU-1 **(B2)**. **(B3)** Representative image for colony formation. **(C1,C2)** Representative image of the EdU assay confirmed that mean density was reduced in PELP1 knockdown group. **(D)** PELP1 knowdown prevented the cell progression from S phase into M phase in cell cycle arrest. The data represent results from one of three independent experiments. **(E1,E2)** Wound healing assay was used to detect the change of migration of cells. The migration ability of AGS was reduced in PELP1 knockdown group. Transwell small chamber assay was used to detect the change of migration and invasion of GC cells. **(F1,F2)** The migration and invasion ability of AGS was reduced in PELP1 knockdown group. **(F3,F4)** The migration and invasion ability of SNU-1 was reduced in PELP1-siRNA group. **(G)** PELP1 silencing inhibited the growth of xenograft in AGS cell inoculated nue mice. Experiments were repeated 3 or 4 times. Values are the mean ± SEM (*p < 0.05, **p < 0.01, ***p < 0.001).

To determine the effect of altered PELP1 expression on GC cell migration, we used siRNA to silence PELP1 expression in AGS and SNU-1cells. Western blot and qRT-PCR results demonstrated that PELP1 expression was significantly inhibited in the PELP1 knockdown group (data not shown). After transfection with siRNA1 of PELP1 into AGS and SNU-1 cells for 48 h, measurements of CCK-8 showed that PELP1 knockdown induced a decrease in the cell proliferation in contrast with the normal control (NC) group by the absorbance (OD) at 450 nm wavelength (Figure 2A, *P* < 0.001). The colony formation assay indicated that silencing of PELP1 inhibited the proliferation of AGS and SNU-1 cells compared with the NC group (Figure 2B). Analysis of images of the EdU assay confirmed that the mean density was reduced in the PELP1 knockdown group (Figure 2C). PELP1 knockdown prevented cell cycle progression from the S phase into the M phase and arrested the cell cycle (Figure 2D). Wound healing assays showed that silencing PELP1 inhibited the migration of AGS and SNU-1 cells (Figure 2E). Furthermore, transwell small chamber assays showed that down-regulation of PELP1 expression attenuated the ability of AGS and SNU-1 cells to migrate and invasion (Figure 2F). Strikingly, PELP1 silencing inhibited growth of nude mice xenograft (Figure 2G). In summary, our data suggest that blockage of PELP1 can inhibit the malignant characteristics of GC cells *in vitro* and *in vivo*.

### PELP1 Activation Promoted Proliferation, Colony Formation, Migration, and Invasion of GES-1 *in vitro*

**Figure 3:**
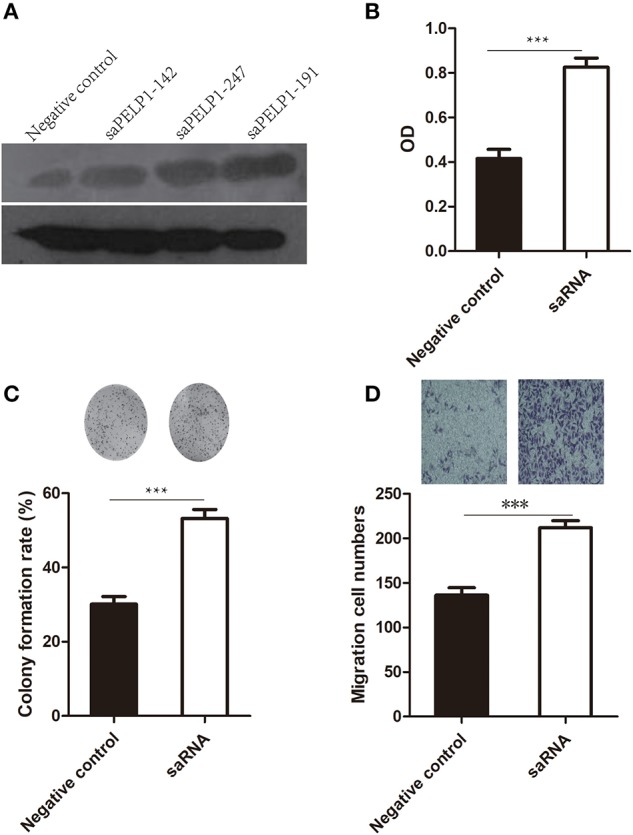
PELP1 activation promoted proliferation, colony formation, migration of GES-1 *in vitro*. **(A)** Representative image of western blot for testing the activation effect of saRNA to PELP1 in GES-1. **(B)** saRNA for PELP1 increased the proliferation ability of GES-1. **(C)** saRNA for PELP1 increased the colony formation ability of GES-1. **(D)** saRNA for PELP1 increased the invasion ability of GES-1. ***p < 0.001.

In view of PELP1 being a potential oncogene, we wonder whether PELP1 can thansform normal gastric epithelium cell. After reinforce PELP1 expression in GES-1, an immortal gastric epithelium cell, it was revealed that the proliferation ability, plate colony formation ability, migration ability of GES-1 were increased (Figure 3). This seemed to suggest that PELP1 has transformation ability in normal gastric epithelium.

### PELP1 Could Directly Target Src, PI3K, and Erk

**Figure 4:**
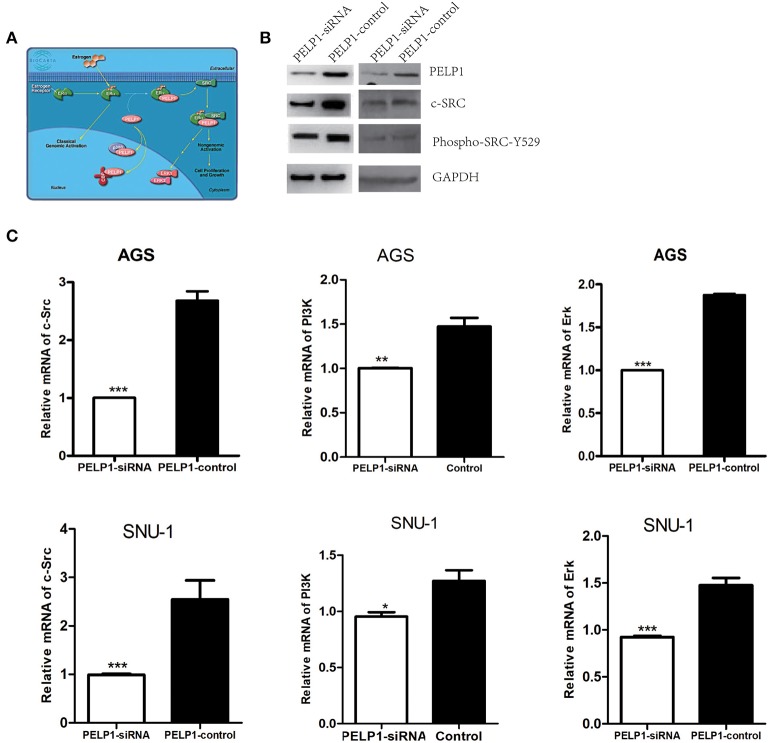
PELP1 knockdown downregulated c-Src-PI3K-Erk pathway. **(A)** BIOCARTA analysis of PELP1 modulation of estrogen receptor activity in human pathway. AGS and SNU-1 cells were transfected with PELP1 siRNA or a non-targeting siRNA (control) for 48 h. **(B)** PELP1 silencing was accompanied by downregulation of c-Src and decreased phospho-Src-Y529 protein as determined by western blot. **(C)** PELP1 silencing was accompanied by the downregulation of c-Src mRNA, PI3K mRNA and Erk mRNA as determined by quantitative RT-PCR. Values are the mean ± SEM (*p < 0.05, **p < 0.01, ***p < 0.001).

We first utilized the Biocarta database to predict PELP1 pathway information in human cells and screened out the Src-Erk pathway which had been found to be a significant regulator of PELP1 modulation of estrogen receptor activity (Figure 4A). Then we focused on the relationship between PELP1 and Src-Erk pathway in GC carcinogenesis. We tested Src-Erk related expression by quantitative RT-PCR and western blot after PELP1 silencing by siRNA in AGS and SNU-1 cells to explore the mechanism of PELP1 downregulation in suppressing GC carcinogenesis. c-Src and phospho-Src were reduced at the protein expression levels (Figure 4B) and c-Src, PI3K, and Erk were reduced at the mRNA expression levels after PELP1 silencing (Figure 4C).

### PELP1 Overexpression Predicted Poorer Clinical Outcomes in GC

**Figure 5:**
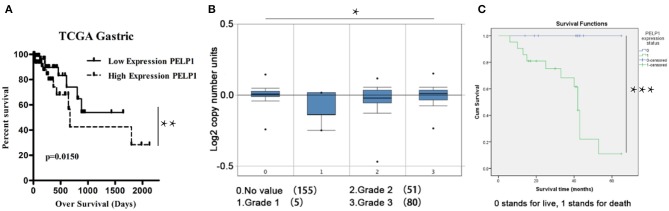
Low PELP1 protein expression is a significant prognostic factor favoring good survival in gastric cancer (GC) patients. GC patients were grouped in 2 categories, those having high and low PELP1 gene expression based on the median. Box plots and data derived from ONCOMINE. Bioinformatics result was partly demonstrated by Kanplan-Meier survival analysis. **(A)** The survival curve analysis showed that there is a obviouly increase in the survival of the patients that have low PELP1 expression. **(B)** Box plots of gastric cancer grade showed that expression of PELP1 is relatively increased in higher grade. Values are the mean ± SEM. **(C)** The data from thirty-six GC tissues microarray revealed that patients with decreased PELP1 showed a longer OS (*p* < 0.05) (*p < 0.05, **p < 0.01, ***p < 0.001).

To determine the relationship between PELP1 expression and prognosis of GC, bioinformatics data from Oncomine were further analyzed. Survival curve results demonstrated that patients with lower PELP1 levels had a better overall survival (OS) than patients with higher PEL1P levels (Figure 5A). In addition, Box plots data further showed that the PELP1 level is correlated with tumor grades of GC patients closely (Figure 5B). Kaplan-meier survival analysis implied high PELP1 expression was a bad prognosis factor in GC (Figure 5C). These final results indicated that PELP1 was an importantprognosis factor in patients with GC; high expression level of PELP1 can be used as an independent biomarker for poor prognosis in patients with GC.

### PELP1 Was a Potential Target of Antipsychotic Drug Chlorpromazine in GC

**Figure 6:**
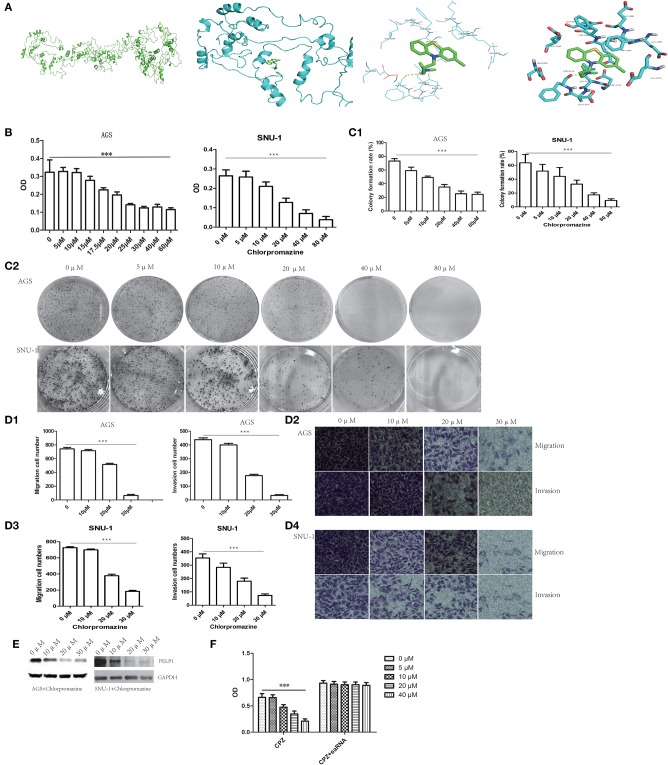
PELP1 can be targeted by chlorpromazine in GC. **(A)** Chlorpromazine interacts with PELP1 in molecular docking model. **(B)** The absorbance (OD) at 450 nm wave-length was reduced in chlorproazine groups in MTT assays dose-dependently. **(C1,C2)** The colony formation ability of AGS and SNU-1 were inhibited with the increasing of CPZ. Transwell small chamber assay revealed CPZ suppressed the migration and invasion ability of AGS **(D1,D2)** and SNU-1 cell **(D3,D4)**. **(E)** CPZ inhibited the protein expression of PELP1 in AGS and SNU-1. **(F)** PELP1 activation compromised the CPZ's inhibitory effect on GC AGS cell. Experiments were repeated 3 or 4 times. Values are the mean ± SEM (***p < 0.001).

Recently, antipsychotic drugs were found to have an anti-cancer effect. Phenothiazine compounds such as thioridazine were demonstrated to have the ability to inhibit some cancers such as melanoma (16), breast cancer (17), and colon cancer (18). There is a little study about chlorpromazine's anti-cancer effect. Luckily, applying molecular docking tool chlorpromazine was combined with PELP1 (Figure 6A). Moreover, we found chlorpromazine can suppress proliferation ability, plate colony formation ability, migration and invasion ability of GC cell line AGS and SNU-1 in a dose-dependent fasion (Figures 6B,C). Moreover, chlorpromazine can downregulate protein expression of PELP1 in a dose-dependent mode (Figure 6D). PELP1 activation by saRNA partly compromised chlorpromazine's inhibitory effection in AGS (Figure 6E). In view of PELP1 is an oncogenic master gene, it was concluded that chlorpromazine inhibited gastric cancer through downregulation of PELP1 and PELP1 may be a molecular therapeutic target in gastric cancer.

## Discussion

Gastric cancer is one of the most common high-mortality malignancies worldwide (19). The pathogenesis of GC is obscure. Emerging evidence suggested all kinds of oncogenes and suppressors were responsible for malignant biology behavior of GC. However, it seems that there was no such a gene which was so strong to be targeted to cure GC. Master genes may have potential to act as such kinds of genes because they located at the key nodes of thousands of pathways. Coregulators were among master genes (20). In this study, we explored the key role of the ER co-regulator PELP1 in GC oncogenesis and its possible underlying mechanisms.

We found that PELP1 not only presents a promoting role in the survival, invasion and metastasis of GC cells, but also participates in the classical signaling pathway. Our results constitute a unique theoretical basis for the pathogenesis and invasiveness of GC.

PELP1 maps to the p13.2 region of chromosome 17 and encodes a protein 1,130 aa in length (21–23). PELP1 is a potential proto-oncogene and functions as a critical ERα coregulatory protein that promotes cancer cells with a distinct growth and survival advantage (24–26).

Initially, PELP1 was reported to be overexpressed in hormone-responsive cancers. For example, it was comfirmed by Cortez et al. that overexpression of PELP1 in the mammary gland contributes to the development of breast cancer using a transgenic mouse model, supporting its carcinogenic potential *in vivo* (27). The carcinogenic signaling of PELP1 is also involved in the progression of other hormone-responsive cancers including breast, endometrial, ovarian and prostate cancer. Astonished, PELP1 can play an oncogenic role in hormone-nonresponsive cancers such as salivary (28), lung (14), pancreas (29), and colon cancer (30), although the function of estrogen receptor α and β in these cancers remained controversial. We speculated oncogenic function of PELP1 in hormone-nonresponsive cancers did not depend on estrogen receptor α and β. In fact, through oncomine data mining and western blot validation, we found ERα and β are low expressed in GC. Maybe there were other mechanism responsible for PELP1 as an oncogene in hormone independent cancers.

In addition, immunohistochemical results from invasive breast biopsies from 1,162 patients demonstrated that PELP1 expression was an independent prognostic predictor of shorter breast cancer-specific survival and disease-free interval, and its expression can be used to assess positive clinical results of breast cancer in ESR1 positive patients (31). Researches have confirmed that PELP1 had a diagnostic indicator for metastatic triple-negative breast cancer, and high expression of PELP1/Ki-67 in tumors was an independent prognostic factor for patients with triple-negative breast cancer (TNBC) (32, 33). Studies using ovarian tumor tissue arrays have shown that PELP1 was overexpressed 2–3-fold in 60% of ovarian tumors (9). PELP1 may play a role in the pathogenesis and progression of astrocytic tumors and may serve as a prognostic biomarker (13). DACH1 expression is dysregulated in human breast cancer and acts as an endogenous inhibitor of ESR1 function. DACH1 is associated with PELP1 as an ESR1 coactivator and the DACH1-PELP1 axis has potential as a biomarker for poor prognosis (34). ESR2 and PELP1 axis are associated with colorectal tumorigenesis and may also have prognostic significance (15). PELP1 has also been identified as one of the potential biomarkers and may play an important role in non-cancer diseases such as asthma attacks in children (35). In our current study, we have demonstrated that PELP1 is carcinogenic in GC from multiple perspectives. In addition, elevated PELP1 expression was positively associated with advanced tumor stage, advanced stage, and lymph node metastasis, consistent with the role of PELP1 identified in other cancers. We also found that histological microarrays showed significant overexpression of PELP1 in GC tissue. Furthermore, we observed that migration and invasiveness were attenuated after silencing of PELP1 expression in GC cell lines, we demonstrated that PELP1 can play a key role in GC migration and invasion.

The pathogenesis of gastric cancer is complicated, and the detailed molecular mechanism remains to be elucidated (36). As a master gene and ER α coregulator, the function of PELP1 is diversified. However, PELP1 excecutives its function irrespective of ER expression status. This point was also proved in our present study. PELP1 is a scaffolding protein that functions as a coregulator of several transcription factors and nuclear receptors. PELP1 can regulate these transcription factors such as activator protein 1 (AP1) (37), nuclear factor κB (NF-κB) (37), signal transducer and activator of transcription (STAT3) (38) and four and a half LIM domains 2 (FHL2) (11) to upregulate downstream target genes. PELP1 also can recruiting chromatin modifier histone deacetylase 2 (HDAC2) to regulate the transcription of target genes (39). Although it has been reported that PELP1 can phosphorylate some key kinases such as c-Src (9), PI3K (40, 41), and ERK (42, 43), it was not shown that PELP1 can phosphorylate PI3K and ERK in GC in our study, but PELP1 silencing inhibited the mRNA transcription of PI3K and ERK in GC, moreover, through oncomine dataminig, it was revealed that many human cancers highly expressed PI3K and ERK in mRNA level (our unpublished data).

Although it has been proved that PELP1 was an oncogene in hormone-dependent cancers and several hormone-independent cancers, it is still unknown whether PELP1 can become a therapeutic target because there is no small molecular chemical or therapeutic monoclonal antibody to target PELP1 nowadays. So it is necessary to clarify which drug can target PELP1. We found classic antipsychotic chlorpromazine, which blocked the dopamine and dopamine receptor pathway to treat schizophrenia (44), can suppress the malignant behavior such as proliferation, plate colony formation, migration, and invasion of gastric cancer dose-dependently. Luckily, it was revealed that chlorpromazine can downregulate the protein expression of PELP1 in gastric cancer dose-dependently, although we did not clarify the exact mechanism for downregulation of PELP1 targeted by chlorpromazine and cannot identify downregulation of PELP1 is the only or major mechanism for chlorpromazine to inhibit gastric cancer. But rescue experiment by saRNA seemed to demonstrate chlorpromazine inhibited GC at least partly through PELP1. We have reason to believe PELP1 is correlated with dopamine receptor because dopamine receptor is a G-protein coupled receptor (45) and PELP1 can interact with these kinds of receptors such as estrogen receptor α functioned in non-genome effect (46), which has been demonstrated in the neuroprotective effect of estrogen (47, 48). PELP1 can function as a scaffold through binding estrogen receptor α and kinases to potentially mediate estrogen-induced kinase signaling. However, till now it is lack about the link in PELP1 and dopamine receptor. As we know, this is the first report about the relationship between chlorpromazine and master gene PELP1.

## Conclusions

In this paper, it was implied PELP1 played an oncogenic role in human gastric cancer induced from *in vitro* cell experiment and clinical sample assay. It was also first demonstrated PELP1 can be regarded as a therapeutic target in gastric cancer, although there is no known small molecular chemical or monoclonal antibody to target or inhibit PELP1. It was firstly implied antipsychotic chlorpromazine can suppress the malignant trait of gastric cancer and significantly downregulate the protein expression of PELP1. Our unpublished data provide more proof to support the notion for Pelp1 as a target of phenothiazine compounds. Molecular docking can effectively dock Pelp1 to phenothiazine compounds. The same or alike phenomena was induced from esophageal cancer, glioblastoma, lung cancer, and liver cancer. In these human cancers, chlorpromazine can effectively inhibit growth, plate colony formation, migration, and invasion and decrease the protein expression of PELP1. In brief, PELP1 is an oncogene in human gastric cancer and the c-Src-PI3K-ERK pathway is upregulated by PELP1. PELP1 can be targeted by phenothiazine compound chlorpromazine and can be regarded as a druggable target.

## Data Availability Statement

All datasets for this study are included in the article/supplementary material.

## Author Contributions

ZN, WX, SL, and FL designed the entire study. HY and ZN wrote the manuscript. HY, YaS, QW, ZW, MH, YuS, and YL performed the experiment. ZN, ZM, and HY analyzed the results and plotted it.

### Conflict of Interest

The authors declare that the research was conducted in the absence of any commercial or financial relationships that could be construed as a potential conflict of interest.

## Abbreviations

GC: gastric cancer
Pelp1: Proline-, glutamic acid-, and leucine-rich protein 1
PI3K: Phosphoinositide 3-kinase
ERK: extracellular signal-regulated kinases
CPZ: chlorpromazine
siRNA: small interference RNA
IHC: immunohistochemistry
SDS: sodium dodecyl sulfate
PCR: Polymerase chain reaction
MTT: dimethyl thiazolyl diphenyl tetrazolium salt
CO_2_: Carbon dioxide
OD: Optical density
ER: estrogen receptor
saRNA: small activating RNA.
